# A Novel Clustering Algorithm for Mobile Ad Hoc Networks Based on Determination of Virtual Links' Weight to Increase Network Stability

**DOI:** 10.1155/2014/432952

**Published:** 2014-04-30

**Authors:** Abbas Karimi, Abbas Afsharfarnia, Faraneh Zarafshan, S. A. R. Al-Haddad

**Affiliations:** ^1^Department of Computer Engineering, Faculty of Engineering, Islamic Azad University, Arak Branch, Arak 38198-38453, Iran; ^2^Department of Computer and Communication Systems Engineering, Faculty of Engineering, UPM, Serdang, Malaysia

## Abstract

The stability of clusters is a serious issue in mobile ad hoc networks. Low stability of clusters may lead to rapid failure of clusters, high energy consumption for reclustering, and decrease in the overall network stability in mobile ad hoc network. In order to improve the stability of clusters, weight-based clustering algorithms are utilized. However, these algorithms only use limited features of the nodes. Thus, they decrease the weight accuracy in determining node's competency and lead to incorrect selection of cluster heads. A new weight-based algorithm presented in this paper not only determines node's weight using its own features, but also considers the direct effect of feature of adjacent nodes. It determines the weight of virtual links between nodes and the effect of the weights on determining node's final weight. By using this strategy, the highest weight is assigned to the best choices for being the cluster heads and the accuracy of nodes selection increases. The performance of new algorithm is analyzed by using computer simulation. The results show that produced clusters have longer lifetime and higher stability. Mathematical simulation shows that this algorithm has high availability in case of failure.

## 1. Introduction

Clustering in mobile ad hoc network (MANET) is defined as the natural arrangement of mobile nodes in numerous different groups [1, 2]. Clustering is used to improve the network's efficiency, ease of navigation, energy consumption, stability, and extending capability [3, 4]. A disadvantage of clustering is the high energy consumption during reclustering operation [5].

A key element in MANETs is the energy consumption of each node [6]. When the energy of a node ends, the node fails. If the failed node is a cluster head, not only the node fails but also all its cluster fails. After a collapse, there are some steps to build a new cluster for the remaining nodes, called as reclustering. Reclustering entails high energy consumption, but the nodes have limited energy resources. Hence, multiple reclustering operations direct the whole network to rapid collapse. Stability of the produced clusters in MANET is a major challenge that clustering algorithms try to resolve. A stable cluster consumes lower energy for transmissions of data between nodes. Consequently, the lifetime of cluster prolongs and fewer reclustering operations are required [7, 8].

Multiple clustering algorithms have been proposed for increasing the stability of clusters in MANET. Weight-based clustering algorithms are the most popular clustering algorithms. They determine the nodes' weights by using limited features of each node. However, it reduces the accuracy of the weights and might lead to selection of an improper node as the cluster head.

In this paper, a clustering algorithm in MANETs is presented. It is called virtual links weight-based clustering (VLWBC). VLWBC determines the weight of every node not only by using the node's own features but also by considering the direct effect of neighbor node's features. This is done through determining weights of virtual links which make the connection between all nodes. Firstly, the weight of virtual links between every two nodes is determined based on four main factors. Then, the final weight of each node is calculated according to the weights of its virtual links. Finally, among multiple nodes and according to their weights a node is selected as the cluster head.

By applying this method, node's mobility, amount of energy, and neighborhood contribution can be predicted in the best way. It also increases the accuracy of generated weights and selects the nodes with the best performance as the cluster heads.

The remainder of the paper is organized as follows.  Section 2 reviews the related literature. The proposed algorithm and its two phases for making clusters and maintaining clusters are presented in Section 3.  Section 4 is devoted to computer and mathematical simulations and their results. Finally, the conclusions are presented in Section 5.

## 2. Related Works

Multiple strategies have been proposed for choosing cluster heads. They choose cluster head based on different factors. The first factor is neighborhood degree. Among the algorithms proposed based on this factor HD (highest degree) [1, 2, 5] and LLC (least cluster change) [1, 2, 5] are the most noticeable. The higher the number of a cluster's members, the lower the clusters, and the lower the number of exchanges between nodes. The disadvantage of this type of algorithms is high energy consumption by cluster heads, as they have numerous members and have to support all of them.

The next proposed factor is node's mobility. MOBIC (mobility based metric for clustering) [1, 2, 5], DDVC (dynamic Doppler velocity clustering) [9], and DLDC (dynamic link duration clustering) [9] are among algorithms proposed based on this factor. In these algorithms a node with the lowest mobility among its neighbors is chosen as the cluster head. If multiple nodes have the same mobility, randomly one of them is chosen as the cluster head. Thus, fewer nodes move out of their clusters and cluster heads are more likely to stay connected to their members. The disadvantage of this type of algorithms is ignoring the other factors, which reduces the stability of the clusters.

The other proposed factor is energy. The algorithms considering energy are “energy efficient clustering algorithms” including SCA (stable cluster algorithm) [1, 2, 5], power-aware connected dominant set [1, 2, 5], and DEECF (stable cluster algorithm energy efficient cluster formation) [10]. In these algorithms, the node with the highest energy among its neighbors is chosen as the cluster head. As a result, the cluster heads benefit the highest energy and have a longer life. Ignoring the other factors is a big disadvantage of this type of algorithms which leads to instability of produced clusters.

The other proposed factor is in fact a mixture of previous factors, that is, neighborhood degree, mobility, and energy. It is load balancing and is also called weight factor. This factor is the best criterion for choosing cluster heads, since it covers all the previous factors. The major combined-metrics-based clustering algorithms are WCA (weight clustering algorithm) [11], DSCAM (distributed scenario-based clustering algorithm for MANETs) [12], EWCA (enhanced weighted clustering algorithm) [13], KCMBC (k-hop compound metric based clustering) [14], CBPMD [15], and MWCA (modified weight clustering algorithm) [16]. In this group of algorithms features of every node are scored and then based on the scores, node's weights are determined. Finally nodes with the highest weights are chosen as the cluster heads. The problem with this type of nodes is that features based on which factors are scored are the same limited features of the node. It decreases the accuracy of produced weights. Hence, the best options may not to be chosen as the cluster heads. The purpose of this paper is to propose an algorithm to solve this problem.

## 3. Virtual Links Weight-Based Clustering Algorithm (VLWBC)

Virtual links weight-based clustering algorithm (VLWBC) determines a weight for each virtual link between nodes. Based on the initial weights, final weights are determined for the nodes. According to final weights, cluster heads are chosen and decide on members of their cluster. It is possible to acquire the final weight of every node based on neighbor nodes' features by determining the weight of links, while with previous methods each node's final weight was determined just by its own limited features. The VLWBC consists of two phases: building the clusters and maintaining the clusters.

### 3.1. Building the Clusters

In order to build a cluster, VLWBC determines virtual links by using

- link neighborhood contribution,
- link stability degree,
- consumed energy by two linked nodes,
- the distance between two linked nodes.

Each node's weight is then determined based on its links' weight. Then, cluster heads are selected based on acquired weights and determine their members.

#### 3.1.1. Link Neighborhood Contribution

The first factor for determining virtual links is the link neighborhood contribution. A node is a nominee to be the cluster head when it has more neighbors. When a node has more neighbors and it becomes a cluster head, the number of exchanges and energy consumption reduce. To include this factor, the neighborhood contribution of a node is calculated. Each node sends a Hello Message to introduce itself to the neighbors. When all messages are sent, each node finds out its neighborhood degree, “*d*,” which shows the number of nodes available in each node's transmission range, |*N*(*v*)|. The neighborhood degree (*d*_*V*_) is given by [11]

(1) $$\begin{array}{c} d_{V}=\left|N\left(v\right)\right|=\underset{v^{\prime }\in V,\;\;v^{\prime }\neq v}{\sum }\left\{\mathrm{dist}\left(v,v^{\prime }\right)<tx_{\text{range}}\right\}. \end{array}$$

Then 1 is divided by the neighborhood degree (*d*_*V*_) to assign equal share of neighborhood contribution to each node. Thus, link's neighborhood contribution percentage is calculated according to

(2) $$\begin{array}{c} P_{V}=\frac{1}{d_{V}}. \end{array}$$

**Figure 1:**
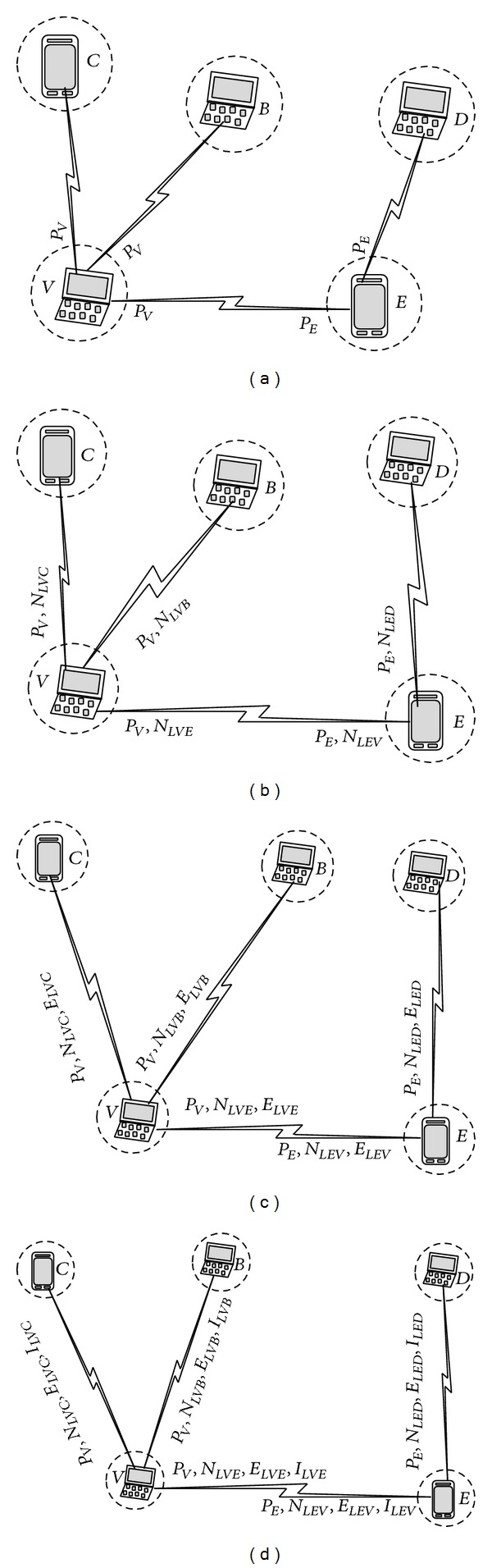
Weight determination of links.

Figure 1(a) shows the neighborhood contribution of *V* and *E* links.

#### 3.1.2. Links Stability Degree

The second factor is the links stability degree. Due to frequent mobility of nodes in MANET, nodes may leave their clusters and make the clusters break down [17, 18]. Therefore, a node needs to have the following qualifications to become a cluster head:

- the least mobility compared to its neighbors: it makes the cluster more stable;
- moving in line with neighbors: it decreases the nodes outward moving and joining other clusters; moreover, message exchange and energy consumption reduce and stability increases;
- the same moving speed as the neighbors:
- a node with these conditions is a candidate to be the cluster head. To determine the node's stability, some information from GPS is required. LET (estimated link duration) which indicates the link duration is calculated and then reversed.

The estimated link duration (LET) [9] is given by

(3) $$\begin{array}{c} \text{LET}=\frac{-\left(ab+cd\right)+\sqrt{\left(a^{2}+c^{2}\right)r^{2}-{\left(ad-bc\right)}^{2}}}{a^{2}+c^{2}}, \end{array}$$

where *i* and *j* are linked nodes. In (3), *a* = *v*_*i*_cos⁡*θ*_*i*_ − *v*_*j*_cos⁡*θ*_*j*_, *b* = *x*_*i*_ − *x*_*j*_, *c* = *v*_*i*_sin*θ*_*i*_ − *v*_*j*_sin*θ*_*j*_, and *d* = *y*_*i*_ − *y*_*j*_ where *v*_*i*_ and *v*_*j*_ are speeds of mobility. This information could be easily acquired through GPS. The result needs to be reversed by

(4) $$\begin{array}{c} N_{L}=e^{-\text{LET}}. \end{array}$$

*N*_*L*_ has reverse relation to stability of a node; that is, a smaller value of this factor shows a more stable link.  Figure 1(b) shows the neighborhood and stability degree of *V* and *E* nodes.

#### 3.1.3. Links Consumed Energy

The third factor is the energy consumption by links. A node with higher energy consumption fails faster [19]. When a cluster head depicts its energy, it fails and also shatters the whole cluster and its links and entails lots of maintenance costs [20]. On the other hand, cluster heads have the highest responsibilities among other nodes. Therefore, they need and consume the highest energy in network and are more likely to drain the energy. As a result, when choosing the clusters their remaining energy needs to be concerned.

Taking this factor into account, the nodes with the highest residual energy and the least consumed energy among their neighbors are selected as the cluster heads [21–23]. The factor (*E*_*L*_) is given by

(5) $$\begin{array}{c} E_{L}=\frac{E_{V}+E_{E}}{2}, \end{array}$$

where *E*_*A*_ and *E*_*V*_ are, respectively, consumed energy of nodes *A* and *V* (Figure 1(c)).

#### 3.1.4. Distance between Two Linked Nodes

The shorter the distance between two nodes is, the less the energy consumption for transferring information between them is. Therefore, for consuming less energy, while transferring information, a node with a shorter distance with its neighbors should be selected as the cluster head [24]. In fact a cluster head with closer distance from its neighbors requires less energy for transferring information which increases the cluster's stability. Parallel K-means clustering algorithm is proposed based on this factor by Thomas and Annappa in 2011 [25, 26]. In this algorithm nodes locate in a cluster with short distances [27, 28]. The distance between a node and its neighbor through its virtual links is calculated in two ways:

1. by receiving the nodes coordinate from GPS,
2. by sending packets and calculating transfer time.

If GPS coordination is utilized, the distance *I*_*L*_ is calculated by

(6) $$\begin{array}{c} I_{L}=\sqrt{{\left(X_{V}-X_{A}\right)}^{2}+{\left(Y_{V}-Y_{A}\right)}^{2}}, \end{array}$$

where (*X*_*V*_, *X*_*A*_) and (*Y*_*V*_, *Y*_*A*_) are coordination of nodes *A* and *V*, respectively. Since connection channels between nodes do not have same bandwidth and speed, the speed factor needs to be included in acquired distance by

(7) $$\begin{array}{c} I_{L}=\frac{I_{L}}{S_{C}}. \end{array}$$

The distance can be calculated by sending packets and measuring the transferring time. Initially, a small packet is sent from one node to other one. A timer is set at the sender node to measure the time duration. The destination sends back the packet once it is received from the origin. The timer is sopped at the origin once the packet is received by the origin. This time duration is called as the transfer time.

Longer transferring time indicates longer distance between the two nodes. The very time could be considered as distance factor (*I*_*L*_). In this method the problem with the first method, that is, channel's speed, does not exist. The acquired *I*_*L*_ is considered as factor 4 (Figure 1(d)).

#### 3.1.5. Links Final Weight

The links final weight (*C*_*L*_) is calculated when the four mentioned factors (*I*_*L*_, *E*_*L*_, *N*_*L*_, and *P*_*V*_) are acquired. Equation (8) calculates the final weight as follows:

(8) CLV=(W1∗PV)+(W2∗NL)+(W3∗EL)+(W4∗IL),

where *W*_1_, *W*_2_, *W*_3_, and *W*_4_ are the weights of each factor and determine the effectiveness of the corresponding factor in the final result on the condition that

(9) $$\begin{array}{c} W_{1}+W_{2}+W_{3}+W_{4}=1. \end{array}$$

**Figure 2:**
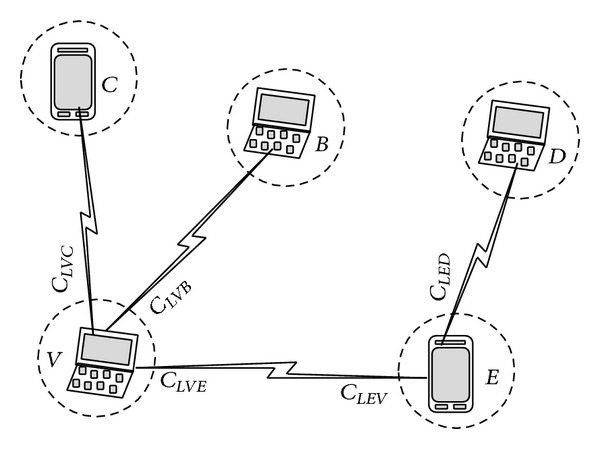
Weight determination for each node.

Weight determination for each node is shown in Figure 2.

#### 3.1.6. Determination of Final Weight for Each Node

In this phase considering the weight of each node's link and load balancing factor, the final weight of nodes is determined by adding up each node's link weight and dividing the result by its neighborhood contribution factor from (10) and (11):

(10) $$\begin{array}{c} C_{V1}=\frac{\sum_{i=1}^{d_{V}}C_{LVi}}{d_{V}}. \end{array}$$

The origin weight should be reversed

(11) $$\begin{array}{c} C_{V1}=e^{-C_{V1}}. \end{array}$$

This factor guarantees the nodes to be distributed fairly among clusters. Without load balancing a cluster may have 2 members, while another has 20 members. In this case, clusters are instable because a cluster has a high load and its cluster head has to spend more energy. Load balancing factor is applied on each cluster head to support only a limited number of nodes and distribute the load fairly among multiple cluster heads.

In order to apply load balancing, a criterion called *δ* is determined. The *δ* represents the optimal number of nodes required to maintain load balancing in a cluster. The difference between the node's neighborhood contribution difference (*d*_*V*_) and a suitable neighborhood contribution criterion (*δ*) is Δ*V*:

(12) $$\begin{array}{c} \Delta_{V}=\left|d_{V}-\delta_{V}\right|. \end{array}$$

A bigger Δ*V* shows a bigger difference between node's neighborhood contributions in an ideal situation. Therefore, the node's final value is smaller. Finally, each node's final weight (*C*_*V*_) is calculated by applying the load factor according to

(13) $$\begin{array}{c} C_{V}=C_{V1}*e^{-\Delta_{V}}. \end{array}$$

#### 3.1.7. Selecting Cluster Heads and Members

After determining each node's final value, nodes send their values to their neighbors. Consequently, all nodes know their neighbors and their own values. A node with the highest value among its neighbors' candidates recognizes itself as the cluster head. Candidates are collected and make a domain set. Member nodes of a domain set send messages to their neighbors and introduce themselves as the cluster heads. In this case one of three different situations occurs for nonmember nodes of a domain set:

1. nonmember nodes of a domain set receive introducing messages from a cluster head. In this case, the node joins the origin cluster;
2. nonmember nodes of a domain set introduce messages from a cluster head. In this case, the free nodes join a cluster head with highest value and introduce themselves to the cluster head as the gateway;
3. nonmember nodes of a domain set receive no message. In this case, a node with the highest value is chosen as the cluster head and others are the members.

**Figure 3:**
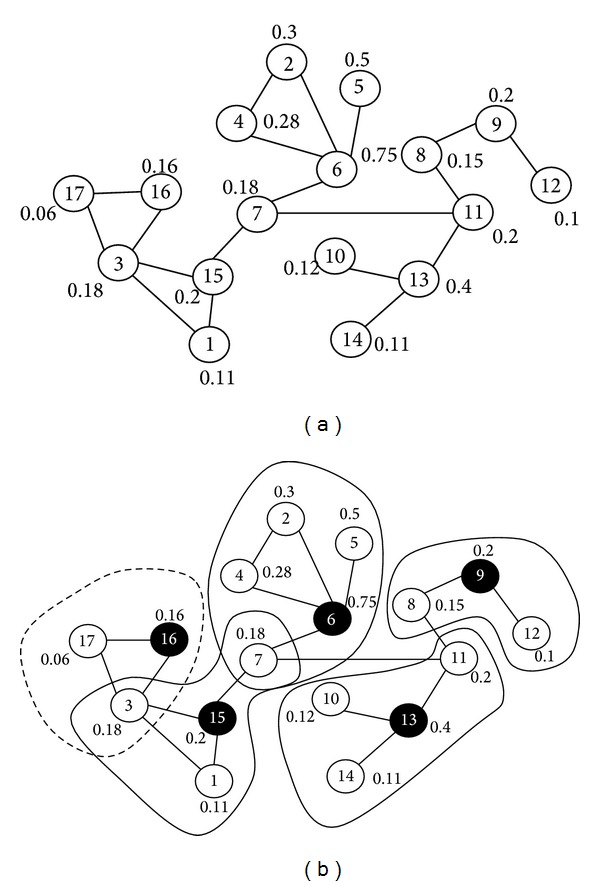
A MANET (a) before clustering and (b) after clustering with VLWBC algorithm.

Figure 3 shows an example of VLWBC algorithm and Table 1 where node 7 receives 2 messages from node 6 and node 15. Node 7 joins node 6 because it has a higher value and introduces itself as the gateway to the cluster head. Nodes 6, 9, 13, and 15 are chosen as cluster heads. Nodes 16 and 17 remain free since they receive no message. Therefore, a node with the higher value, that is, node 16, is chosen as the cluster head and node 17 joins the cluster head.

### 3.2. Clusters' Maintenance Phase

This phase starts immediately after creation of the first cluster. This phase is activated when one of the following problems occurs. VLWBC algorithm has a solution for each problem.

1. A node leaves its cluster range: in this case, the node needs to join a new cluster. The node that left the cluster sends a message to cluster heads in its transmission range. When a cluster head is ready to accept the node, it sends back a message and declares its value. By comparing received values from multiple clusters (if any), the node joins a cluster with the highest value.
2. Cluster head fails as a result of discharged battery: in this case, two actions are possible: (a) nodes join the other cluster heads through the process explained in part 1; (b) choosing a new cluster head among survived nodes and inviting other nodes to the new cluster.
3. Member nodes fail as a result of discharged batteries: in this case, the cluster head removes dead nodes from its members list. Then, it sends messages to member nodes and checks for their effectiveness and other changed features. If no message is received back from a node, the node is either dead or out of cluster's range. Therefore, the node is removed from the cluster's members list.
4. Cluster heads interfere: in this case, values of two interfered cluster heads are compared and the one with higher value is chosen as the cluster head. Then, the nodes of the lower valued cluster head join the new cluster. Among nodes which are not members of the new cluster head will be chosen based on their values and others will join it.

## 4. Simulation

The proposed algorithm has been investigated in two methods: computer simulation and mathematical simulation. The resulting charts and graphs will be discussed in the following sections.

### 4.1. Computer Simulation

To study the effectiveness of VLWBC algorithm several tests are performed by NS-2.34 simulator [29]. Nodes of a mobile ad hoc network are distributed in a 2000∗2000 meter environment. The nodes bandwidth is 2 Mbps/sec. Maximum size of transmitted packets is 512 bytes and an access protocol to channel MAC 802.11 CSMA/CA has been predicted. Batteries of nodes are initially full and the ideal limit for the load setting factor is considered as 8 nodes. Further information on simulation setup is available in Table 2.

In simulation tests, the algorithm has been compared with three popular algorithms including LEACH (low-energy adaptive clustering hierarchy) [30–32], MWCA (modified weighted clustering algorithm) [16], and WCA (weighted clustering algorithm) [11].

First, the clusters' lifetime which indicates their stability is calculated. Then, the consumed energy of the network's nodes is investigated and at the end the number of clustering and reclustering operations is evaluated. Finally, the effectiveness of the algorithm and the stability of produced clusters are compared to other algorithms.

All factors have been investigated based on transmission rate variation. Increasing the nodes' transmission range increases the number of neighbors. Therefore, each node is able to cover a larger range. As the number of nodes is important in choosing cluster heads, any change in transmission range will affect the other factors.

#### 4.1.1. Clusters Lifetime

Lifetime is defined as the time duration from selection of a certain node as the cluster head until its death. The longer the lifetime is, the more effective the network is. Clusters lifetime is expressed in seconds and highly depends on the way that the clusters are chosen by clustering algorithm.

**Figure 4:**
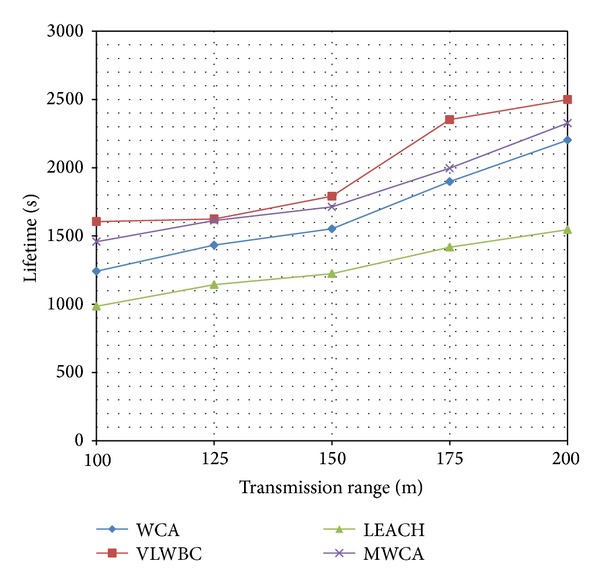
Lifetime factor as the nodes' transmission range increases.

Table 2 represents simulation features and clusters' lifetime based on transmission range variations.  Figure 4 shows the acquired results from the simulation.

Figure 4 shows that VLWBC algorithm has a longer time compared to the other three algorithms. The reason for improvement of lifetime is selection of proper clusters by VLWBC algorithm, whereas in WCA and MWCA the competency of nodes is acquired through information from links between the nodes and MWCA. The node's weight is calculated only through the node's limited features. It makes the weights not chosen carefully for being the cluster heads. Meanwhile, load setting factor which is applied to the proposed algorithm has made the algorithm able to transfer less information in a network communication. Hence, it saves more energy and increases the network lifetime.  Table 3 shows the difference of lifetime in two areas of the graph.

As it is obvious from Table 3, there is an increase in transmission range; hence, the number of neighbors grows. Thus, the lifetime of clusters dramatically increases with VLWBC algorithm compared to MWCA algorithm. This improvement reaches 17% in some points and ultimately presents improvement of VLWBC algorithm in the stability of the network. This is due to the number of neighbors and increasing the effect of their features. Therefore, weights can be calculated precisely and the load factor clusters can be created with a fair number of members. If the transmission range and the number of the neighbors are low and nodes cover only a small area, the number of clusters' members might be unfair and load balancing is not carried out precisely.

#### 4.1.2. Consumed Energy

Consumed energy by the node for transferring information during a specific time period is called network's consumed energy. The lower the amount of consumed energy in the nodes, the longer the lifetime of the nodes and the more stable the clusters. This value is expressed in term of mj.

**Figure 5:**
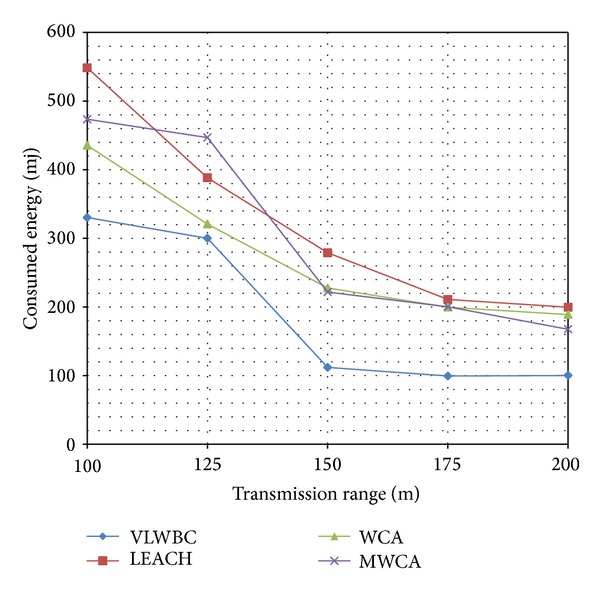
Consumed energy with increase in transmission rate.

Table 2 shows features of simulation. Consumed energy is calculated based on variation of transmission range.  Figure 5 is a diagram obtained from these experiments.

It is obvious from Figure 5 that a lower energy has been consumed in VLWBC algorithm compared to the other algorithms. At starting point WCA algorithm shows a good performance, but as the transmission range increases, it leads to a lower performance. The reason is that the energy consumption by the nodes has direct effect on determining the node's weight in the proposed algorithm. Furthermore, the cluster heads and their member nodes are chosen with optimal distance. Therefore, they can transfer information with minimal energy dissipation.

Since load balancing factor has not been applied in MWCA algorithm, the number of members of the cluster heads is not fair and there is no balance in the distribution of nodes among the cluster heads. Consequently, the energy consumption is high in MWCA algorithm.  Table 4 shows a comparison in two areas of the graph and the difference of consumed energy.

Table 4 represents the energy consumed in VLWBC algorithm compared to other algorithms. It shows that VLWBC improved energy efficiency up to 50% and helped in stabilizing the network. Furthermore, when the number of neighbors is high and the nodes can transfer the information directly to a long distance, less energy is consumed. In VLWBC algorithm the reduction rate of consumed energy is much higher compared to the other algorithms. It leads to a big difference in energy consumption between proposed algorithm and others when the transfer range is 175.

#### 4.1.3. The Number of Clustering and Reclustering Operations

All clustering algorithms try to improve the number of clustering and reclustering operations. When a cluster is dead, the reclustering phase starts by the survived nodes to choose a node as the cluster head. Selected cluster head creates a new cluster or bounds survived nodes to other clusters. This procedure consumes too much energy, leads to clusters' death, and reduces the network's stability. Considering this fact, a fewer clustering phases yield to a more stable network. Furthermore, as the number of clusters decreases, the number of transformed messages between clusters and the energy dissipation decrease.

**Figure 6:**
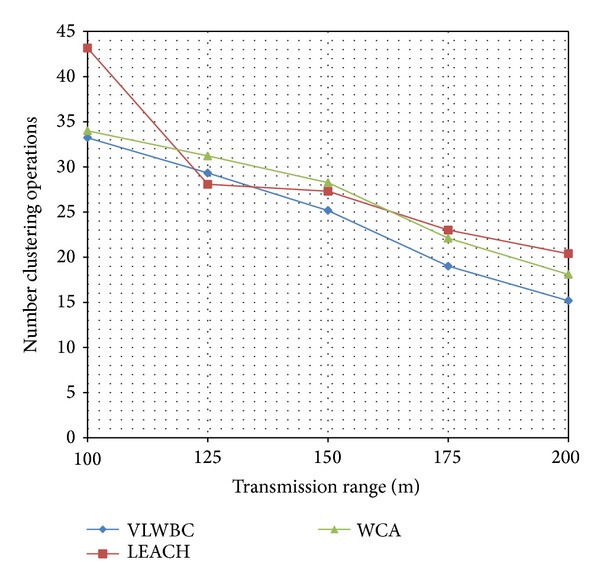
The number of clustering and reclustering operations with different range.

The number of clustering and reclustering operations has been shown in Figure 6 for different transmission range. In VLWBC algorithm fewer clustering and reclustering operations have occurred. They become even lower by increasing the transfer range.

The number of clustering and reclustering operations has been improved because it considered the links' weights stability degree as an important factor. This factor predicts nodes' mobility and causes some nodes to have the highest weights and to be chosen as the cluster heads. These nodes have the highest similarity in mobility to their neighbors. When the cluster heads have similar mobility to their neighbors, for a longer time the nodes stay in their clusters and cluster heads stay in their collections. Thus, the network requires fewer operations for maintenance and reclustering. On the other hand, clusters' maintenance phase has been designed in such a way that when a cluster fails, a new cluster is chosen properly.

Table 5 shows a comparison performed in 2 areas of the graph and the differences in the number of produced clusters.

As seen in Table 5 the number of reclustering operations in VLWBC algorithm has improved compared to the other algorithms. The improvement is up to 19% in some points which leads to higher stability of the network in VLWBC algorithm. Moreover, small number of produced clusters and fewer transferred messages are obtained for higher transfer ranges.

Considering the experiments by computer simulations and analysis performed on the resulted graphs, it can be concluded that VLWBC algorithm improves the stability of the network compared to other algorithms. It proves the high efficiency of VLWBCalgorithm.

### 4.2. Mathematical Simulation

Markov model (Markov process model) [33] is utilized for availability analysis of VLWBC versus WCA (weighted clustering algorithm) [11] and DDVC (dynamic Doppler velocity clustering) [9] algorithms.

The availability of proposed algorithm is utilized for analysis of the algorithms. Algorithm availability is the probability of the algorithm to remain available and efficient when there is certain failure for a certain time. Due to some reasons, some phases of the algorithm may not be fulfilled temporarily and the algorithm has to continue without the results of that phase. For instance, to calculate links' stability degree, information from GPS is required. If GPS is not available on a certain time, the calculation phase of links' stability degree could not be carried out completely. Therefore, the algorithm has to determine the weights and complete the clustering without results from this phase.

**Figure 7:**
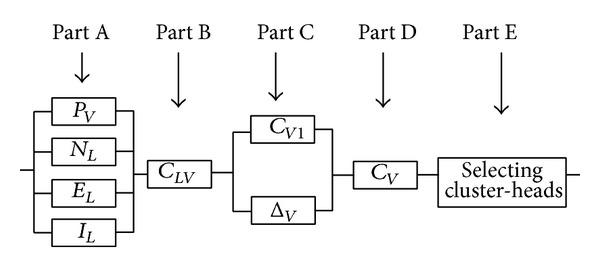
VLWBC parallel-series model.

The availability along with other parameters from computer simulation shows the algorithm's efficiency in different conditions. In this study, availability is varied between 0 and 10 and is acquired by time variations. For calculating the availability of an algorithm, algorithm's different states are determined. For this purpose the parallel-series model of VLWBC is delineated in Figure 7. This model shows the way algorithm's states switch and establishes the output and input of each phase [34].

From Figure 7, the VLWBC algorithm has one of the states as follows.

- State 0: system works properly.
- State 1: a component of part A is broken.
- State 2: two components of part A are broken.
- State 3: three components of part A are broken.
- State 4: all components of part A are broken.
- State 5: components of part B are broken.
- State 6: the second component (Δ_*V*_) of part C is broken.
- State 7: the first component (*c*_*V*1_) is broken.
- State 8: all components of part C are broken.
- State 9: all components of part D or E are broken.

**Figure 8:**
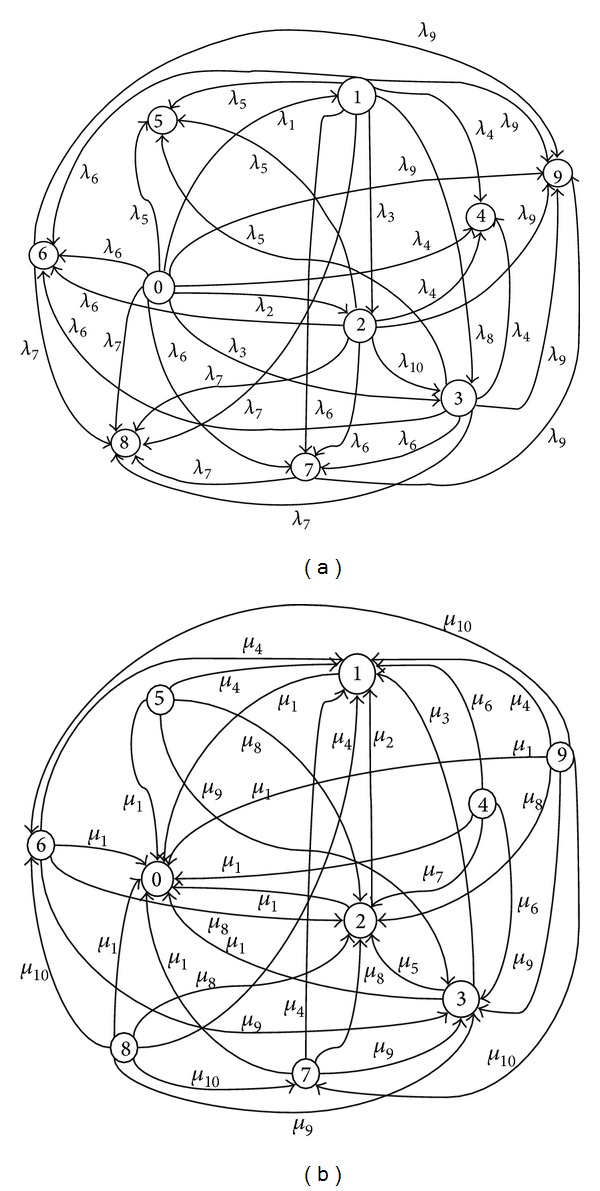
Markov model of VLWBC algorithm with determination of (a) failure rate (*λ*) and (b) repair rate (*μ*) in each path.

To determine the current state of the algorithm in case of failure of components, state diagram of Figure 8 is drawn. A component's failure rate is *λ* and its repair rate is *μ*.

*λ*s and *μ*s are acquired based on state variation diagram as follows:

(14) $$\begin{array}{c} \lambda_{1}=4\lambda \Delta t,\;\;\lambda_{2}=7\lambda \Delta t,\;\;\lambda_{3}=4\lambda \Delta t,\\ \lambda_{4}=1\lambda \Delta t,\;\;\lambda_{5}=1\lambda \Delta t,\;\;\lambda_{6}=1\lambda \Delta t,\\ \lambda_{7}=1\lambda \Delta t,\;\;\lambda_{8}=3\lambda \Delta t,\;\;\lambda_{9}=1\lambda \Delta t,\\ \lambda_{10}=2\lambda \Delta t\\ \mu_{1}=1\lambda \Delta t,\;\;\mu_{2}=2\lambda \Delta t,\;\;\mu_{3}=3\lambda \Delta t,\\ \mu_{4}=1\lambda \Delta t,\;\;\mu_{5}=2\lambda \Delta t,\;\;\mu_{6}=4\lambda \Delta t,\\ \mu_{7}=6\lambda \Delta t,\;\;\mu_{8}=1\lambda \Delta t,\;\;\mu_{9}=1\lambda \Delta t,\\ \mu_{10}=1\lambda \Delta t. \end{array}$$

The algorithm works properly in states 0, 1, 2, 3, and 6. Therefore, the system's availability is given by

(15) AS=P0+P1+P2+P3+P6=1−(P4+P5+P7+P8+P9).

For calculating the availability, the occurrence probability of states needs to be obtained. *λ*s and *μ*s are substituted and Δ_*t*_ is divided by the two sides to acquire the main probability. Then, it is turned into Laplace transforms. As an example *P*_0_ would be like

(16) P0′(t)=−(19λ)P0(t)+1λP1(t)+1λP2(t)+1λP3(t) +1λP4(t)+1λP5(t)+1λP6(t)+1λP7(t)+1λP8(t) +1λP9(t).

Then, Laplace inverse is obtained from acquired probability. By using the obtained values, system availability function is calculated. Mathematica 6.0 is used to draw the graphs [35]. All calculations are done by Mathematica 6.0 as well.

**Figure 9:**
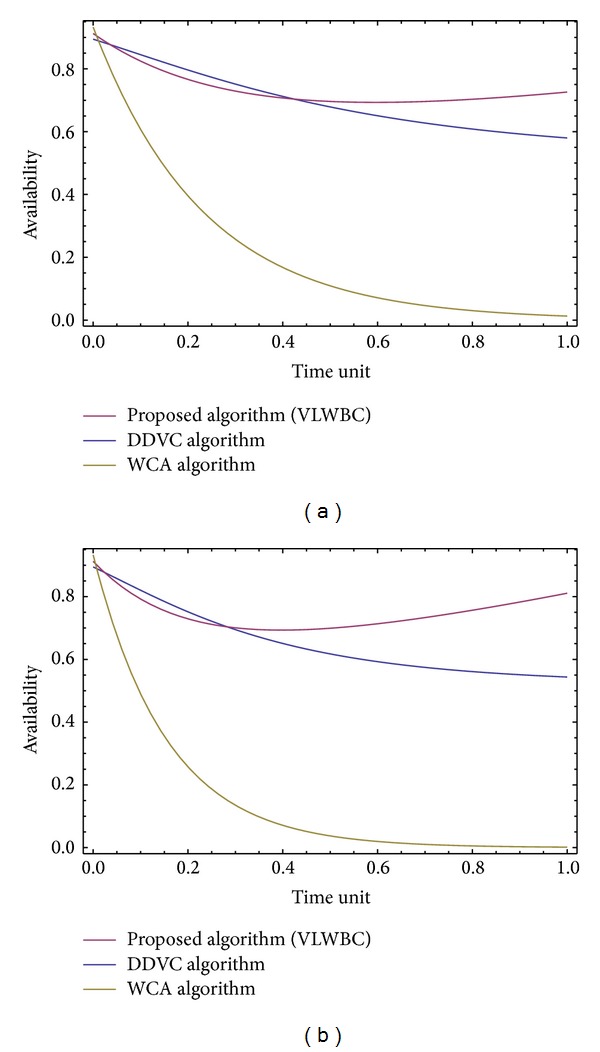
VLWBC algorithm availability when (a) *λ* = 0.2 and (b) *λ* = 0.4.

Availability graphs of VLWBC are drawn in Figure 9, where *λ* = 0.2 and 0.4 and *t* = [0.1,…, 1]. DDVC algorithm was proposed by Sakhaee and Jamalipour in 2008 [9] and WCA algorithm was proposed by Chatterjee et al. in 2002 [11]. Obtained graphs show that VLWBC has a higher availability compared to WCA and DDVC algorithms. On the other hand, WCA loses its availability over time, while availability of VLWBC algorithm improves. DDVC's availability is initially high and even higher than VLWBC but it decreases over time. In our proposed algorithm the availability improves over time and never decreases. The reason is using a large number of components which cover each other in case of failure; for example, there are four components in the first phase, while one of them is enough to continue the clustering operation. However, the last component of WCA has a high probability of failure which degrades the availability of WCA algorithm to zero over time.

From the experiments and the obtained graphs, the improvement of availability of the VLWBC algorithm can be calculated compared to the other two algorithms. The results are shown in Tables 6 and 7.

It can be concluded from Tables 6 and 7 that not only the availability of VLWBC algorithm is better than the other two algorithms but also their difference increases over time; for example, the availability of VLWBC algorithm at time unit of 0.6 and failure rate of 0.2 is 0.69 which shows 6.16% improvement compared to DDVC algorithm. This is because the phases of the proposed algorithm have been determined in a suitable way. Therefore, if some phases of the algorithm fail to work properly, the algorithm can continue to work with a high probability.

Considering the obtained results from mathematical simulation, it could be stated that the availability of the proposed algorithm is higher than the other two algorithms. Advantage of VLWBC algorithm is that the algorithm might be unavailable very rarely and works properly most of the time.

## 5. Conclusions

VLWBC algorithm has been proposed for clustering MANETs. The virtual links' weight for every node is calculated and based on links' weight the nodes' final weight is determined. By determining links' weight and calculating each node's weight, a node's weight is acquired according to the features of the neighbor nodes. Four factors including energy, stability, neighborhood contribution, and the link's length are used. For the final value of each node, values of its links and load balancing factor have been used.

Considering computer simulation and analysis on obtained graphs, it could be concluded that the VLWBC algorithm improved the network's stability, increased the lifetime of the clusters, and decreased the consumed energy. Mathematical simulations showed that the availability of VLWBC algorithm was improved. VLWBC had the higher availability in comparison with other methods and continued to function with a high probability when some components are not available.

## Figures and Tables

**Table 1:** The domain set for Figure 3(a).

Node weight	Node number
0.75	6
0.2	9
0.4	13
0.2	15

**Table 2:** Simulation parameters in NS2 simulator.

Parameter	Meaning	Value
*N*	Number of nodes	100–500
*X*∗*Y*	Size of network	2000∗2000 m
*S*_range	Speed range	0–5 m/s—randomly
*T*_range	Transmission range	100–200 m
Run time	Time of simulation	600 s
*W*_1_, *W*_2_, *W*_3_, and *W*_4_	Weights	0.25, 0.25, 0.25, and 0.25

**Table 3:** Comparison of lifetime factors for proposed algorithm.

Algorithm	Lifetime with 150 transmission range	Lifetime with 175 transmission range
VLWBC	1789 s	2353 s
MWCA	1616 s	2000 s
Increment %	10.70%	17.65%

**Table 4:** Comparing the proposed algorithm and WCA algorithm in terms of energy consumption.

Algorithm	Consumed energy with 150 transmission range	Consumed energy with 175 transmission range
VLWBC	112 mj	100 mj
WCA	225 mj	200 mj
Decrement %	50.22%	50.00%

**Table 5:** Comparison of clustering and reclustering operations.

Algorithm	Number of reclustering operations with 140 transmission range	Number of reclustering operations with 175 transmission range
VLWBC	26.5	18.9
LEACH	27.2	23.4
Decrement %	2.57%	19.23%

**Table 6:** VLWBC availability improvement compared to DDVC.

Parameter	*λ* = 0.2, time unit = 0.6	*λ* = 0.4, time unit = 0.4
VLWBC algorithm	0.690058	0.694818
DDVC algorithm	0.649989	0.649960
Improvement %	6.164565	6.901655

**Table 7:** VLWBC availability improvement compared to WCA.

Parameter	*λ* = 0.4, time unit = 0.6	*λ* = 0.2, time unit = 0.8
VLWBC algorithm	0.7598708	0.709807
WCA algorithm	0.0156789	0.035085
Improvement %	4746.4547	1923.1967
